# Differences in immune responses to *Haemonchus contortus* infection in the susceptible Ile de France and the resistant Santa Ines sheep under different anthelmintic treatments regimens

**DOI:** 10.1186/s13567-019-0722-3

**Published:** 2019-11-29

**Authors:** Ana Cláudia A. Albuquerque, Cesar Cristiano Bassetto, Fabiana A. Almeida, Katie A. Hildersley, Tom N. McNeilly, Collette Britton, Alessandro F. T. Amarante

**Affiliations:** 1 School of Veterinary Medicine and Animal Science, UNESP–São Paulo State University, Rua Professor Doutor Walter Mauricio Correa s/n, Botucatu, SP 18618-681 Brazil; 2 Institute of Biosciences, UNESP–São Paulo State University, Rua Professor Dr. Antônio Celso Wagner Zanin, 250, Botucatu, SP 18618-689 Brazil; 3 Institute of Biodiversity, Animal Health and Comparative Medicine, UoG–University of Glasgow, Glasgow, G61 1QH UK; 4 Disease Control Division, MRI –Moredun Research Institute, Pentlands Science Park, Bush Loan, Penicuik, Midlothian, EH26 0PZ UK

## Abstract

Understanding the immunological basis of resistance to gastrointestinal nematode infections in livestock is important in order to develop novel methods of parasite control such as vaccination or genetic selection for parasite resistance. The present study aimed to investigate differences in immune response between parasite resistant Santa Ines and susceptible Ile de France sheep breeds to natural *Haemonchus contortus* infection. Parasitological parameters, humoral immunity, local and circulating cellular immune responses were evaluated in 19 Santa Ines and 19 Ile de France lambs undergoing different anthelmintic treatments regimens: suppressive treatments (SUP) or targeted selective treatments (TST) over a 5-month grazing period. Santa Ines lambs had significantly lower *Haemonchus* faecal egg count and worm burden compared to Ile de France regardless of treatment regime. In addition, circulating blood eosinophils count and parasite-specific IgG levels were significantly higher and more rapidly induced in Santa Ines lambs. Abomasal immune responses were generally greater in the resistant breed, which had significantly higher levels of parasite-specific IgA in mucus, and elevated number of globule leukocytes and CD3+ T cells within the abomasal mucosal. Furthermore, numbers of POU2F3+ epithelial cells, a tuft-cell specific transcription factor, were also elevated in the Santa Ines breed, suggesting that this breed is better able to initiate T-helper type 2 immune responses within the abomasum. In conclusion, the differential immunological responses detailed here are relevant to understanding resistance to gastrointestinal nematodes in other host breeds, as well as to resistance breeding as a sustainable control approach for parasitic infections.

## Introduction

Gastrointestinal nematode (GIN) infections are among the main health problems affecting ruminants and are responsible for huge economic loss to the livestock industry [1]. Control of GIN is heavily dependent on anthelmintic treatment [2], however the high frequency of dosing increases the prevalence of anthelmintic-resistant nematode populations, even with new compounds, such as monepantel [3, 4]. Insight into mechanisms involved in the appropriate gastrointestinal immune response to GIN is fundamental for the development of sustainable approaches, such as selective breeding and vaccination to reduce anthelmintic use [5–11].

*Haemonchus contortus* is an important GIN in sheep husbandry across the humid temperate and tropical regions worldwide. It is a highly pathogenic, blood-feeding parasite, responsible for massive economic loss, due to reduced productive performance, compromised reproduction and high mortality [3, 7, 12]. The emergence of multi-drug resistance *H. contortus* has focussed research on development of alternative control strategies, such as selective breeding for animals resistant to infection [5] and vaccination [8]. A vaccine, Barbervax^®^, comprising native proteins extracted from the nematode gut has been introduced in Australia [13] and tested with promising results in Brazil [14–16]. However, its widespread use is limited, mainly due to cost, licensing, requirement for multiple vaccinations and additional strategies, such as nutritional improvement [15].

Host resistance to nematode species is related to the ability to develop strong innate and acquired immunity, limiting the establishment of infective larvae and/or eliminating the worm population [9, 17–21]. This differs from resilience, defined as the ability of animals to produce and reproduce in the face of parasite infection [22]. Resistance against GIN infection is a moderately heritable trait, and selection of animals with an efficient immune response to GIN may increase flock resistance and potentially be exploited as an alternative control measure [3, 23].

Protection against GIN infection is mediated by type 2 immune responses, involving induction of cytokines and antibody production, and expansion and mobilization of innate and adaptive immune cells [6, 10, 11, 24]. Parasite clearance is associated with innate and adaptive responses characterised by mucosal mast cells hyperplasia, globular leukocyte appearance, increased eosinophil concentration and induction of goblet cell hyperplasia with mucus production, while humoral responses involve IgG, IgE and IgA production [23, 25, 26]. However, the mechanisms involved in the initiation and the development of the immune response to GIN have not been fully elucidated. Some studies indicated that in the gut mucosa of resistant animals, upregulation of a T helper 2 (Th2)-type response is responsible for protection, while in susceptible animals, with chronic nematode infections, a Th1-type response is enhanced [19, 27, 28].

More recently, it was demonstrated in murine models that epithelial cells called tuft cells might be responsible for initiating type 2 responses to nematode infection, and release IL-25 to stimulate IL-4 production by Th2 cells through a feed-forward loop involving Group 2 Innate Lymphoid Cells (ILC2) [29, 30]. Wild-type mice with experimental *Nippostrongylus brasiliensis* infection showed worm clearance 13 days after infection. In contrast, mice deficient in tuft cells, through knockout of tuft cell specific transcription factor POU2F3, presented numerous worms 42 days post-infection. This demonstrated the importance of tuft cells in the immune response against GIN in mice [29]. Our ongoing work is detailing the dynamics during infection and gene expression profile of ovine abomasal POU2F3+ cells to determine if these cells are equivalent to mouse tuft cells (Hildersley et al. unpublished data).

Our previous work identified Santa Ines hair sheep as naturally resistant to *H. contortus* infections at different age categories, in which they presented a high number of blood eosinophils, high production of IgG, low eggs per gram of faeces (EPG), normal packed cell volume (PCV) and low worm burden, in addition to requiring fewer anthelmintic treatments in comparison with other commercial wool-sheep breeds [26, 31]. The present study aimed to elucidate and compare the innate and adaptive immunological response between Santa Ines and Ile de France lambs. Humoral and cellular responses were compared during infection and at post-mortem to determine in detail the mechanisms of resistance against natural *H. contortus* infection in the field.

## Materials and methods

### Ethical considerations

The experiment was carried out at São Paulo State University (UNESP), Botucatu-SP, Brazil. All animal procedures were in accordance with the ethical standards and were approved by the Animal Use Ethics Committee of the FMVZ/UNESP (47/2016).

### Experimental design

The experimental design was described previously [4] and was carried out over 5 months, from October 2016 to February 2017, during the rainy season. Briefly, the experiment was in a 2 × 2 factorial design, with 19 Santa Ines (SI) and 19 Ile de France (IF) 3-month-old uncastrated male lambs purchased from commercial farms located in São Paulo state, Brazil. The lambs were raised indoors at their farms of origin. The lambs were allocated to the two anthelmintic treatment groups: suppressive (SUP)—animals drenched every 2 weeks with monepantel (2.5 mg/kg; Zolvix^®^, Novartis) in order to maintain the grazing lambs and their pastures as free of worms as possible; targeted selective treatment (TST)—animals under continuous challenge infection for 5 months and underwent monepantel treatment only when they showed a PCV ≤ 20% corresponding to category 3 of FAMACHA© method [32], in order to prevent the occurrence of mortality and/or severe haemonchosis. Animals were allocated into each group by stratified randomization, balanced as far as possible taking into consideration body weight and nematode faecal egg counts (FEC) at start of the trial prior to drenching.

Suppressive groups (SUP-SI and SUP-IF) were drenched on arrival with the following combination of anthelmintics: albendazole (10 mg/kg, Endazole^®^ 10 Cobalto, Hipra), levamisole (9.4 mg/kg, Ripercol^®^ L 150 F, Zoetis), and monepantel (2.5 mg/kg, Zolvix^®^, Novartis). Afterwards, they were allocated to the paddock after no nematode eggs were detected in a series of faecal examinations. The last anthelmintic drench for lambs under suppressive treatment was administered on week 19 and they were slaughtered on week 21. The lambs in the TST group (TST-SI and TST-IF) were not treated on arrival at the experimental facilities. Instead, they were introduced onto clean pasture that became contaminated with eggs from the nematodes acquired at their origin farm.

### Faecal examination

Faecal samples were collected upon animals’ arrival and then weekly, directly from the rectum of each animal in polyethylene bags previously labelled and kept refrigerated until processing. The modified McMaster technique [33] was used to measure faecal eggs count (FEC), in which each nematode egg counted represented 100 eggs/g of faeces (EPG). Faecal cultures were prepared separately for each group for production of GIN infective third-stage larvae (L3) on animals’ arrival and then weekly to identified morphologically and counted [33]. Based on the proportion of L3 identified as *H. contortus* in the cultures, the *Haemonchus* EPG of each lamb was estimated.

### Worm burden

All lambs were slaughtered and the gastrointestinal tract removed. The abomasum was opened along its greater curvature and the contents placed in graduated buckets. Aliquots of 10% of the total abomasum were collected individually, stored in plastic flasks and preserved in −20 °C freezer. All nematodes present in the 10% preserved material were identified morphologically and quantified, according to their developmental stage [33, 34] and stored in 70% ethanol.

### Haematology

Blood samples (5 mL) were collected weekly by jugular vein puncture into Vacutainer^®^ tubes containing anticoagulant (EDTA). The blood in the tube was centrifuged to allow plasma separation. Aliquots of plasma samples were stored at −20 °C and −80 °C prior to ELISA. Eosinophil counts in the peripheral blood were performed in a Neubauer’s chamber after staining with Carpentier’s solution [35], and the counts expressed as the number of eosinophil cells per µL of blood.

### Histology

Abomasal tissue samples were fixed in 4% buffered formaldehyde for 48 h, then moved to 70% ethanol, and paraffin-embedded. Tissue sections were cut to 5 µm thick and mounted on glass slides. Eosinophils and globule leukocytes were counted on Haematoxylin and Eosin (H&E) stained sections whereas mast cells were counted on sections stained with 1% toluidine blue. All cells were counted in sixty randomly selected fields of view per animal in a 0.01 mm^2^ area at 1000× magnification [adapted from [6]. The counts were expressed as cells/mm^2^ tissue surface.

### Immunohistochemistry (IHC)

Immunohistochemistry sections were mounted on Superfrost Plus glass slides (Thermo Fisher Scientific). The tissues were dewaxed in xylene, rehydrated and heat-induced antigen-retrieval was performed by autoclaving at 121 °C for 10 min in 10 mM citrate buffer pH 6.0. This was followed by two washes in PBS and one in PBS/0.5% Tween 80 (PBS/T80) for 5 min, and then endogenous peroxidase activity was blocked with 0.3% hydrogen peroxide in PBS/T80 for 20 min at room temperature (RT). Following a further two PBS/T80 washes for 5 min each, slides were loaded into a Sequenza immunostaining centre rack (Thermo Fisher Scientific). The sections were incubated with 25% goat serum in PBS/T80 for 1 h at RT to reduce nonspecific binding and background staining. The primary antibodies and their appropriate isotype control were diluted in PBS/T80 containing 10% normal goat serum (NGS) and incubated overnight at 4 °C. Slides were washed twice in PBS and incubated with the secondary antibody at RT for 30 min. This was followed by PBS wash and incubation with 3,3′-diaminobenzidine (DAB) for 7.5 min at RT. Thereafter, the slides were washed in distilled water, counterstained with haematoxylin, dehydrated and mounted in Shandon synthetic mountant (Thermo Fisher Scientific). Detailed information of all antibodies used in immunohistochemistry are specified in Additional file 1.

Images were taken from 10 random areas of each slide for POU2F3+ (putative tuft cell) counting at 400× magnification using microscope Olympus BX50 with digital camera Olympus DP70. POU2F3+ epithelial cell frequency was calculated as the proportion of epithelial cells positive for POU2F3, expressed as a percentage (adapted from [30]). Ten random areas of each slide were used to perform the T and B cell counting using calibrated graticule at 250× magnification on Dialux 20 EB Microscope (Leitz Wetzlar, Germany). T and B cells counts were expressed as the number of positive cells per mm^2^.

### Enzyme-linked immunosorbent assay (ELISA)

#### Haemonchus contortus-specific IgG

Plasma samples collected at 11 time-points were used to determine IgG levels against L3-soluble extract of *H. contortus.* The L3 extract was prepared as previously described [17]. A previously described protocol was applied to determine the parasite-specific plasma IgG levels [36], with some modifications: the plates were coated with 2 µg of antigen/mL; each wash was done three times, rotating through 180 and re-washing three more times; the negative control (NC) sample used was from a worm free animal, as previously described [37]; the plasma positive control (PC) sample used was from a sheep artificially infected with both *H. contortus* and *Trichostrongylus colubriformis* every 3 days for 84 days. Results were expressed as the percentage of optical density (OD) value of the PC plasma sample [38].

#### Haemonchus contortus-specific IgA

A 5 cm piece from the greater curvature of abomasum was sampled and stored at −20 °C until processing for the extraction of mucus. Tissues were thawed and mucus was scraped off with a glass slide and stored in a falcon tube on ice, followed by addition of PBS supplemented with protease inhibitors (Complete^®^, Roche) to each sample in a proportion of 4:1 (4 mL PBS + 1 mL of mucus). The samples were shaken for 1 h at 4 °C and centrifuged for 30 min at 4 °C and 3000 × *g*. The supernatant was collected and centrifuged again for 30 min at 4 °C and 15 000 ×* g.*

ELISA assays for parasite-specific IgA recovered from abomasal mucous were carried out against *H. contortus* L3 and adult extract as previously described [36] with a few differences: plates were coated with 5 µg of antigen/mL; each wash was done three times, rotating through 180° and re-washing three more times; mucus samples were diluted in PBS-GT (1:20) and rabbit anti-sheep IgA peroxidase-conjugated antibody was diluted at 1:20 000. The results were expressed as OD value minus OD-blank sample [38].

#### Total plasma IgE

Total IgE antibody levels were determined in plasma from six time-point samples by sandwich enzyme immunoassay using a Sheep Immunoglobulin E ELISA kit (My BioSource©, San Diego, USA). The data were expressed in µg/mL.

### Statistical analysis

All data were submitted to normality test and transformed using log_10_ (x + 1) when necessary. Data with single measures and data for repeated measures at several time points were analysed by ANOVA using the General Linear Model (GLM) and groups means were compared by Tukey’s test using Statistical Analysis System, version 9.2 (SAS Institute, Inc., Cary, NC, USA). Values of *P* < 0.05 were considered statistically significant.

## Results

### Parasitology

**Figure 1:**
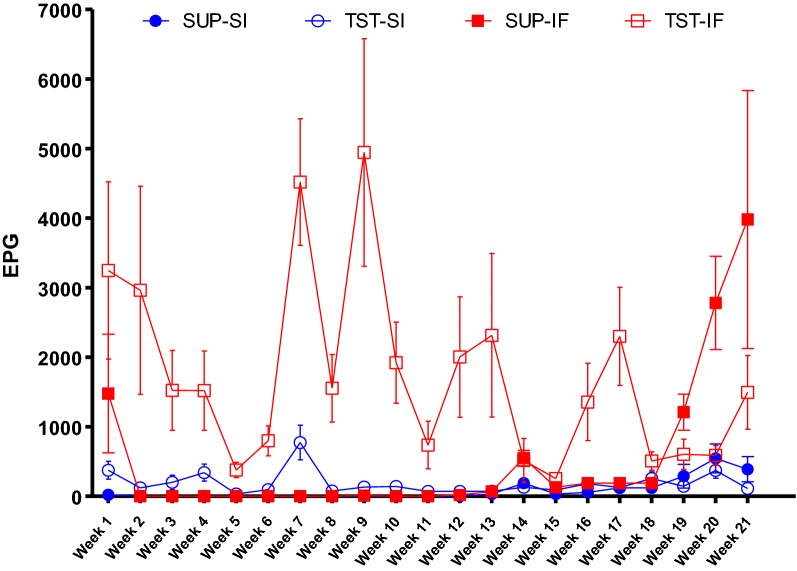
**Means of** ***Haemonchus contortus*** **eggs per gram of faeces (EPG).** EPG counting of the Santa Ines (SI) and Ile de France (IF) lambs naturally infected with *H. contortus* and under suppressive (SUP) or targeted selective treatment (TST) with anthelmintics. Values represent mean ± standard error.

Before the beginning of the trial, the young lambs had been infected with *Haemonchus* spp. and *Trichostrongylus* spp. on their farms of origin. Then, during the trial, the lambs under suppressive treatment were maintained with low exposure of strongyle infection during the first 4 months of the trial, with increase in EPG values and L3 detected in the faecal culture in the last month of the trial, due to development of anthelmintic resistance [4]. The animals under TST were continuously challenged by natural GIN infections for 5 months as determined by the presence of parasite eggs within faeces throughout the study period (Figure 1). *Haecmonchus contortus* at different developmental stages, was the only species found in the abomasum at post-mortem, with significantly lower worm burdens in the Santa Ines lambs in both suppressive and TST treated groups (*P* < 0.0001, Table 1).

**Table 1:** **Developmental stages of** ***Haemonchus contortus*** **means (minimum–maximum values) of the Santa Ines and Ile de France lambs under suppressive or targeted selective treatment (TST) programme with anthelmintics**

Development stages	Santa Ines		Ile de France		Effects (*P* value)	
	Suppressive (*n* = 9)	TST (*n* = 10)	Suppressive (*n* = 10)	TST (*n* = 9)	Breed	Treatment programme
Early L4	31 (0–80)	114 (0–650)	141 (30–600)	597 (10–142)	< 0.0001	ns
Female late L4	38 (0–120)	60 (0–360)	103 (0–360)	414 (0–1450)	0.0036	ns
Male late L4	20 (0–70)	63 (0–360)	100 (10–420)	456 (0–2010)	0.0002	ns
Female early L5	92 (0–590)	5 (0–30)	389 (100–830)	179 (0–460)	< 0.0001	0.0094
Male early L5	49 (0–270)	16 (0–140)	334 (80–710)	160 (0–530)	< 0.0001	0.0066
Adult female	121 (0–400)	17 (0–110)	921 (210–2620)	279 (0–600)	< 0.0001	0.0015
Adult male	121 (0–420)	8 (0–50)	881 (190–2190)	289 (0–740)	< 0.0001	0.0011
Total worm burden	472 (20–1330)	283 (0–1680)	2869 (720–7100)	2373 (60–7040)	< 0.0001	0.0125

L: larvae, ns: not significant (*P* > 0.05).

Mean total *H. contortus* burden was lower in TST groups of both breeds, suggesting that continuous infection for 5 months induced some protection in the TST-lambs in comparison with lambs undergoing suppressive anthelmintic treatments, in which patent GIN infections were only seen at the end of the trial (Table 1), related to the development of anthelmintic resistance in surviving worms [4]. There was a breed effect on all worm stages of development (Table 1), with Santa Ines lambs presenting a lower burden for all stages recovered (*P* < 0.0001). No significant breed × treatment interaction was identified in *H. contortus* burdens. The intestinal GIN species *T. colubriformis*, *Strongyloides papillosus*, *Cooperia curticei* and *Trichuris* spp. were also found in the lambs of the both breeds.

There was a substantial difference between the breeds in the number of salvage drenches required in the TST groups: all Ile de France lambs needed to be treated with monepantel due to PCV < 20%, treatments ranging from one to four times, totalling 19 drenches during the trial. In contrast, only two Santa Ines lambs needed to be treated: one once and another twice, detailed in [4]. One of these Santa Ines presented elevated *Haemonchus* worm burden (1680 specimens) in relation to animals in the same group (group average = 283). Therefore, our results showed that besides a significant breed difference, there was also genetic variability in each breed influencing the degree of resistance.

### Antibody responses

#### Plasma IgG and IgE levels

**Figure 2:**
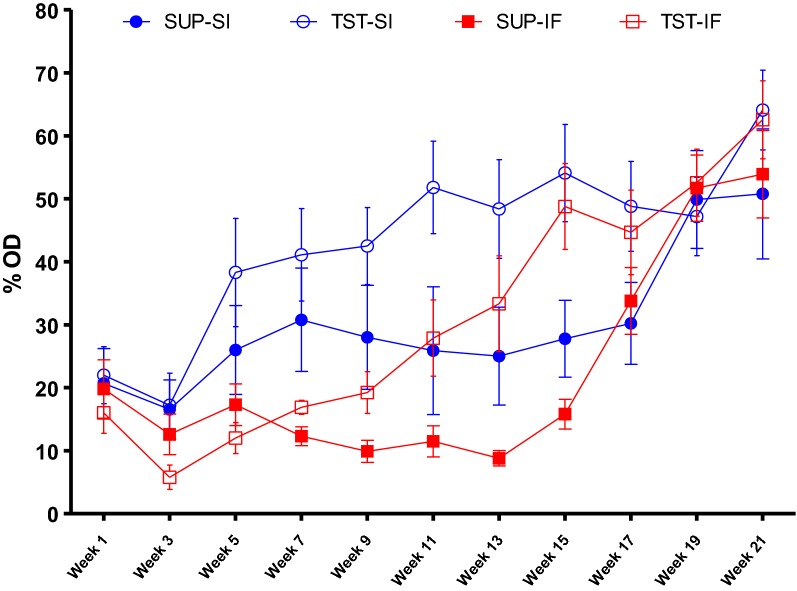
**Mean levels of anti-*Haemonchus contortus*** **L3 IgG (% OD) in plasma.** IgG levels of the Santa Ines (SI) and Ile de France (IF) lambs naturally infected with *H. contortus* and under suppressive (SUP) or targeted selective treatment (TST) with anthelmintics. Values represent mean ± standard error.

**Figure 3:**
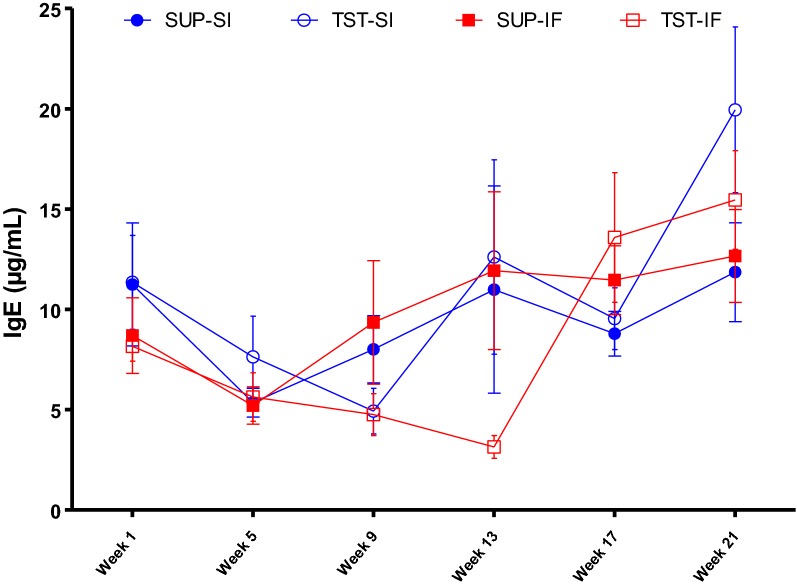
**Total IgE plasma concentration (µg/mL).** IgE concentration of the Santa Ines (SI) and Ile de France (IF) lambs naturally infected with *H. contortus* and under suppressive (SUP) or targeted selective treatment (TST) with anthelmintics. Values represent mean ± standard error.

The mean levels of anti-*H. contortus* L3 IgG in plasma showed changes over time, resulting in significant time × breed (*P* < 0.04) and time × treatment (*P* < 0.0001) interactions. Breed had an effect (*P* < 0.05) on mean anti-*H. contortus* IgG levels in the beginning of the trial (on week 5, week 7 and week 9), when Santa Ines lambs showed a sharp increase in production of anti-*H. contortus* IgG level. Treatment program had an effect on IgG levels at five time-points, between week 9 and week 17, when TST groups showed the highest IgG levels. In the last month of the trial, the means of all groups were similar (Figure 2). In contrast, we did not detect any breed or treatment effect (*P* > 0.05) in total IgE levels in plasma. There was a significant time × treatment interaction (*P* < 0.02), with the highest mean IgE levels in groups under TST treatment at the last sampling (Figure 3).

#### Abomasal IgA levels

**Figure 4:**
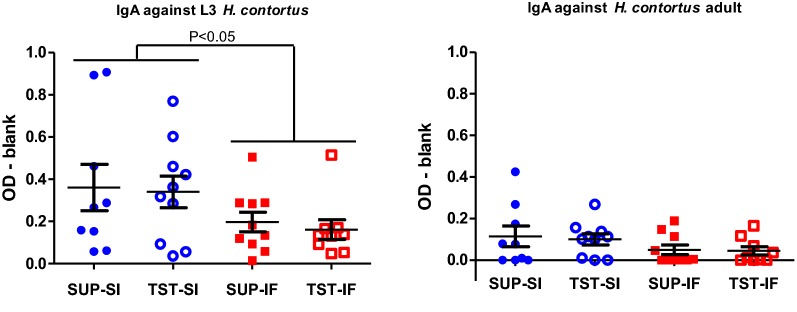
**Mean levels of anti-*Haemonchus contortus*** **L3 and adult—specific IgA (OD—blank).** IgA levels measured in the abomasal mucus collected post mortem of the Santa Ines (SI) and Ile de France (IF) lambs naturally infected with *H. contortus* and under suppressive (SUP) or targeted selective treatment (TST) with anthelmintics. Values represent mean ± standard error.

There was a significant breed effect on mean levels of *H. contortus* L3-specific IgA in abomasal mucus. Levels of L3-specific IgA were significantly (*P* < 0.05) higher in Santa Ines lambs compared to Ile de France lambs independent of treatment group (Figure 4). Mean levels of adult *H. contortus*-specific IgA were relatively low and similar among the four groups.

### Cellular responses

#### Blood eosinophils

**Figure 5:**
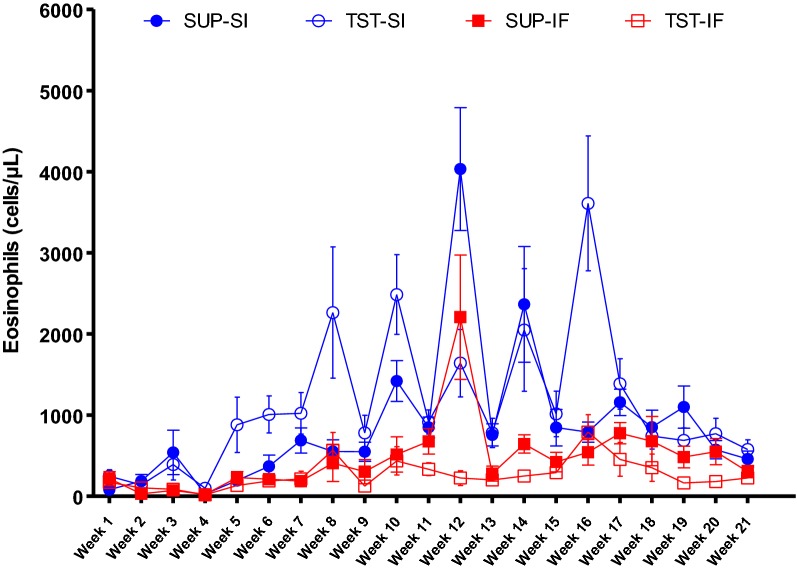
**Mean blood eosinophil count (cells/µL).** Blood eosinophil counting of the Santa Ines (SI) and Ile de France (IF) lambs naturally infected with *H. contortus* and under suppressive (SUP) or targeted selective treatment (TST) with anthelmintics. Values represent mean ± standard error.

The mean blood eosinophil count of each animal showed changes over time with significant time × breed (*P* < 0.02) and time × treatment interactions (*P* < 0.0001). There was breed x treatment interaction at three time-points at the beginning of the trial: week 4, week 5 and week 6, when TST-SI lambs had significantly higher averages than the other groups. Breed had the greatest effect on mean blood eosinophil count, with Santa Ines lambs showing early eosinophilia and the highest mean eosinophil counts during the trial. All means increased progressively until week 16, followed by stabilization until the end of the trial (Figure 5).

#### Abomasal cellular immune responses

Santa Ines and Ile de France lambs under TST showed the highest number of mast cells (MC) and globular leukocytes (GL). In the case of GL, but not MC, there was a significant (*P* < 0.05) effect of breed, with Ile de France under suppressive treatment displaying the lowest GL counts. A significant effect of treatment was found for both cells (*P* = 0.0001), with higher MC and GL counts in lambs undergoing TST. A significant breed x treatment interaction was also found for GL counts. Numbers of eosinophils were similar among groups without any breed or treatment effect (*P* > 0.05) (Table 2).

**Table 2:** **Averages (minimum–maximum values) of eosinophils (cells/mm**^**2**^**), mast cells (cells/mm**^**2**^**), globule leukocytes (cells/mm**^**2**^**), POU2F3 + cells (%), T cells (cells/mm**^**2**^**) and B cells (cells/mm**^**2**^**) in abomasum mucosa of the Santa Ines and Ile de France under suppressive or targeted selective treatment (TST) programme with anthelmintics**

Cells	Santa Ines		Ile de France		Effects (*P*-value)		
	Suppressive (*n* = 9)	TST (*n* = 10)	Suppressive (*n* = 10)	TST (*n* = 9)	Breed	Treatment programme	Breed × treatment
Mast cells	51 (17–97)	132 (17–305)	28 (0–83)	113 (15–165)	ns	0.0001	ns
Globule leukocyte	66 (27–145)b	148 (55–252)b	30 (0–118)a	142 (18–238)b	0.0031	< 0.0001	0.0228
Eosinophils	95 (10–277)	90 (5–197)	80 (5–317)	42 (7–60)	ns	ns	ns
POU2F3+ cells	7.33 (3.0–12.0)	11.18 (1.9–13.7)	3.64 (2.0–5.5)	7.62 (4.6–11.3)	< .0001	< .0001	ns
T cells	993.5 (768–1499)	1102.5 (740–1458)	808.0 (481–1167)	788.0 (582–1030)	0.0012	ns	ns
B cells	94.0 (27.2–181.7)	124.5 (50.9–249.4)	99.2 (14.8–260.9)	45.4 (12.8–130.4)	ns	ns	ns

Means followed by different letter in the same line differ from each other by Tukey’s test (*P* < 0.05).

ns: not significant (*P* > 0.05).

**Figure 6:**
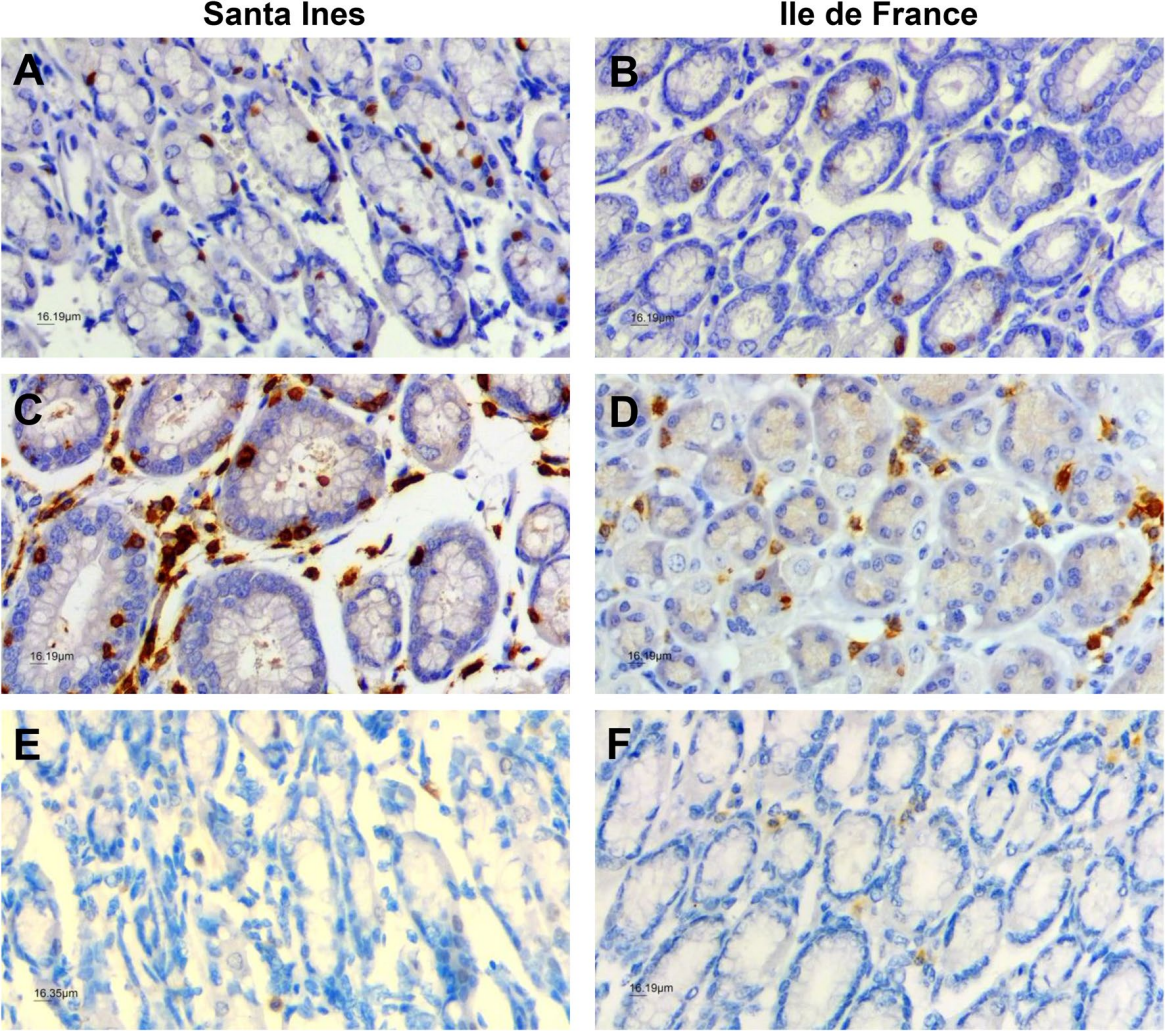
**Immunohistochemical labelling of abomasum mucosa sections showing positive cells. A**, **B** POU2F3+ cells; **C**, **D** CD3+ T cells; **E**, **F** CD79α+ B cells (×400 magnification). The micrographics **A**, **C**, **E** show tissue from the Santa Ines lamb with no detectable *Haemonchus contortus* worm; while **B**, **D**, **F** show tissue from Ile de France lamb with a *H. contortus* burden of 1860.

IHC revealed positive nuclear staining in the abomasum mucosa of Ile de France and Santa Ines lambs of POU2F3+ epithelial cells (Figures 6A and B) and positive labelling of CD3+ T cells (Figures 6C and D), and CD79α+ B cells (Figures 6E and F). There was a significant breed effect (*P* < 0.05) on % POU2F3+ cells in the epithelium and T cell numbers within the abomasal mucosa, with highest mean levels in the Santa Ines breed. The treatment regimen had an effect (*P* < 0.05), with greater frequency of POU2F3+ cells in the TST groups. B cell numbers were similar among groups (*P* > 0.05) (Table 2).

## Discussion

The immune response against GIN infection is characterised by reductions in faecal egg count and worm burden and can be influenced by several factors such as age, sex, physiological status (especially parturition and lactation), and breed [26, 39–41]. Previous studies have shown that Santa Ines is a relatively resistant breed to *H. contortus* infections at different stages of life: sucking lambs [42], weaned lambs [26], ewes around parturition and during lactation [39]. However, the mechanisms involved in their greater resistance to GIN infection are not clear.

Taking into consideration that resistance to parasites is largely immune mediated [13, 28], our aim was to look for differences in immune responses that could explain resistance or susceptibility between breeds. The infections were naturally acquired by the lambs during grazing. Therefore, it was not possible to determine the number of L3 they ingested during the trial. Nevertheless, we assume that, in each treatment group, animals ingested a similar number of infective larvae because they were under the same grazing management. Our study was divided into two stages: firstly, the evaluation of circulating immune responses in lambs exposed to natural *H. contortus* infection and secondly, the evaluation of local abomasal cellular and antibody responses from the same lambs at post-mortem.

In the first stage, the major differences were higher levels of anti-*H. contortus* L3-specific IgG and number of blood eosinophils in the Santa Ines breed, especially at the beginning of the trial, indicating that the response against *H. contortus* occurs earlier in this breed than in the Ile de France. At post-mortem, there were marked differences between breeds, with Santa Ines showing significantly higher levels of mucus anti-L3 IgA, POU2F3+ cells, T cells and GL. These local responses may be implicated in limiting establishment of infective larvae and/or eliminating adult parasites [6, 11, 20, 30, 44].

Studies of other resistant sheep breeds identified earlier and robust initial type 2 responses against GIN infection, with production of specific antibodies [10, 19, 20, 27]. In the current study, earlier and marked IgG production were observed in Santa Ines lambs, however, both breeds presented similar IgG levels at the end of experiment, demonstrating that with the progression of the trial, the Ile de France lambs were also responding to *Haemonchus* infection. Such level of IgG response, however, was not enough to protect the Ile de France from haemonchosis. Similar results have been reported previously, with no difference in IgG level between resistant and susceptible breeds infected with *H. contortus* [27], suggesting that resistance to *H. contortus* infection is not related to plasma IgG level.

IgE is the main antibody involved in the type 2 response against GIN infection and its expression is associated with greater resistance and shows moderate to high heritability [23]. We observed that total IgE concentration increased over time, however, there was no significant difference between breeds. This may be due to IgE being present at extremely low serum concentrations with a short half-life. Most of the IgE is found irreversibly attached to specific receptors of mast cells and eosinophils [45, 46]. In future studies, it will be interesting to evaluate IgE attached to inflammatory cells or *H. contortus*-specific IgE level in the blood serum, which can provide more informative results.

In the present study, the resistant breed presented the highest means of mucosal anti-*H. contortus* L3-specific IgA, suggesting that this immunoglobulin had an important regulatory role in the local immune response against *H. contortus* infection in the Santa Ines lambs. IgA is associated with reductions in worm length and fertility in *H. contortus* infection, and early worm expulsion [44, 47, 48].

The early and robust blood eosinophil expansion in the Santa Ines lambs for almost the entire experimental period may contribute to low nematode establishment and elimination [49], and as consequence low FEC and low worm burden in the resistant breed. The magnitude of eosinophil response may be related to resistance to helminth infection. This cell type remains briefly in the bloodstream before migrating to tissues, where they may stay for several weeks, depending on the presence of Th2 cytokines, or undergo apoptosis [49, 50]. Once triggered, eosinophil degranulation may occur by exocytosis, piecemeal degranulation, explosive degranulation with consequent cellular lysis or formation of extracellular traps [51–53]. Unlike in blood, numbers of eosinophils in the mucosa were similar in the two breeds. This may reflect the time-point at which mucosal eosinophil were enumerated, as at post-mortem circulating levels of eosinophils were similar between the two breeds. Another possibility is that the turn-over rate of mucosal eosinophil populations in the Santa Ines breed was more rapid, meaning that higher recruitment of blood eosinophils into the abomasum in the Santa Ines breed would not necessarily result in elevated eosinophil numbers in the mucosa. Enumeration of mucosal eosinophils at time-points of infection where blood eosinophil numbers were significantly different between the two breeds would be required to investigate this further.

Rapid rejection of nematodes in sheep is associated with expansion and mobilization of MC and GL into the gut mucosa [6, 54, 55]. The strength of MC response is linked to Th2 cytokines and IgE production [56, 57], whereby resistant breeds show greater responses [20, 27, 43]. We observed higher MC and GL cells counts in the Santa Ines resistant breed, but only the GL count was significantly different between breeds. GL derive are thought to be de-granulated MC [54]. Previous study using a gut washing method has shown that GL may leave the epithelium and can be found in the gut lumen in large amounts [58]. Future studies could apply this method, which may identify an even more marked difference between resistant and susceptible animals.

Epithelial cells are the first cell type in contact with the parasite, indicating that before immune engagement there is a need for epithelial activation [59]. Epithelial chemosensory cells called tuft cells were shown to drive early signals in the small intestine to initiate type 2 immunity against helminth infection in mice [10, 60]. These cells are the dominant source of IL-25 that induces a Th2 response through the action of group 2 innate lymphoid cells (ILC2s) to produce specific type 2 cytokines [61, 62]. At present we cannot be sure that the POU2F3+ cells identified here are the equivalent of tuft cells in mice, and work is ongoing to determine this (Hildersley et al. unpublished data). Interestingly, Santa Ines sheep showed a higher percentage of POU2F3+ cells in the abomasum mucosa compared to Ile de France, indicating a possible role for these cells in resistance against *H. contortus* infection. The B cells count was similar between breeds, in agreement with other studies [6, 27], that reported no difference in B cells between resistant and susceptible breeds.

There was a marked difference between treatments groups with regard to MC, GL counts and POU2F3+ cells. The greater number of these cells in the TST groups of both breeds was possibly a result of the longer and greater GIN challenge in comparison with animals under the suppressive treatment, which showed increase in parasite egg counts in the last month of the trial and had significantly higher *H. contortus* worm burdens at post-mortem. This suggests that expansion and/or recruitment of these Th2 associated immune cells may be most sensitive to nematode continuous challenging compared to other immune responses measured in this study.

Assessing differences in the local cellular responses between breeds at early time points after infection could be informative. Particular focus would be on T cells and POU2F3+ cells (putative tuft cells), and whether earlier and/or greater activation of these putative tuft cells drive a greater type 2 response in Santa Ines breed. Determining the resistance mechanisms is important for understanding breed resistance and identifying genetic markers that may advance selective breeding as a sustainable control strategy [5].

In conclusion, Santa Ines lambs showed early and robust immune response against *H. contortus* infection, with strong local immune responses, effective in limiting the establishment and/or eliminating the worm population. This breed has potential value in improving the resistance of sheep to GIN infection, as a sustainable alternative control strategy.

## Supplementary information

**Additional file 1. Antibodies used in immunohistochemistry to identify positive cells on the abomasum mucosa.**
