# Supplementary material for: Differences in immune responses to Haemonchus contortus infection in the susceptible Ile de France and the resistant Santa Ines sheep under different anthelmintic treatments regimens
Source: Vet Res. 2019 Nov 29;50:104. doi: 10.1186/s13567-019-0722-3 (PMC6884896; doi:10.1186/s13567-019-0722-3)
Supplement: Supplementary file 1 — Additional file 1. Antibodies used in immunohistochemistry to identify positive cells on the abomasum mucosa. [file 13567_2019_722_MOESM1_ESM.docx]

**Additional file 1: Antibodies used in immunohistochemistry to identify positive cells on the abomasum mucosa**.

| Antibody | Specificity | Specification | Dilution | Isotype control | Secondary | Primary antibody supplier |
| --- | --- | --- | --- | --- | --- | --- |
| Polyclonal rabbit anti-human POU2F3 | Putative tuft cell | HPA019652 | 1:500 | rabbit IgG | anti-rabbit polymer-HRP | Sigma-Aldrich, St. Louis, MO, USA |
| Polyclonal rabbit anti-human CD3 | T cells | A0452 | 1:200 | rabbit IgG | anti-rabbit polymer-HRP | Dako, Kyoto, Japan |
| Monoclonal mouse anti-human CD79α | B cell | JCB117 | 1:600 | mouse IgG1 | anti-mouse polymer-HRP | Dako, Carpinteria, CA, USA |
